# Endothelium and Its Alterations in Cardiovascular Diseases: Life Style Intervention

**DOI:** 10.1155/2014/801896

**Published:** 2014-02-26

**Authors:** Gaia Favero, Corrado Paganelli, Barbara Buffoli, Luigi Fabrizio Rodella, Rita Rezzani

**Affiliations:** ^1^Section of Anatomy and Physiopathology, Department of Clinical and Experimental Sciences, University of Brescia, Viale Europa 11, 25123 Brescia, Italy; ^2^Department of Medical and Surgical Specialties, Radiological Sciences and Public Health, University of Brescia, Viale Europa 11, 25123 Brescia, Italy

## Abstract

The endothelium, which forms the inner cellular lining of blood vessels and lymphatics, is a highly metabolically active organ that is involved in many physiopathological processes, including the control of vasomotor tone, barrier function, leukocyte adhesion, and trafficking and inflammation. In this review, we summarized and described the following: (i) endothelial cell function in physiological conditions and (ii) endothelial cell activation and dysfunction in the main cardiovascular diseases (such as atherosclerosis, and hypertension) and to diabetes, cigarette smoking, and aging physiological process. Finally, we presented the currently available evidence that supports the beneficial effects of physical activity and various dietary compounds on endothelial functions.

## 1. Introduction

The term endothelium was coined in the 1865 by the anatomist Wilhelm His, who differentiated the inner lining of body cavities from epithelium. Wilhelm His defined as endothelium the cell lining blood vessels, lymphatics, and mesothelial-lined cavities. This definition was later modified including only the inner cell stratum of blood vessels and lymphatics. Actually, the endothelium is considered a monocellular layer that separates all tissues from the circulating blood [1, 2]. In the 1950s and 1960s, the use of electron microscopy provided detailed and innovative ultrastructural information, revealing in endothelial cells (ECs) the presence of characteristic organelles, such as plasmalemmal vesicles, successively defined caveolae, and Weibel-Palade bodies [3, 4], and showed that the ECs are characterized by structural and functional heterogeneity. In fact, the shape and organization of cells vary across the vascular tree. Blood vessel endothelium traverses each and every tissue, but each vascular bed has unique structural and functional properties and this reflected ECs heterogeneity [5].

ECs are absent in invertebrates, cephalochordates, and tunicates but are present in the three major groups of extant vertebrates: hagfish, lampreys, and jawed vertebrates. This observation underlines that the endothelium is shared by jawless and jawed vertebrates and that it was present in the ancestor of these animals. So, it is a tissue structure conserved during the evolution of vertebrate. Yano et al. [6], for the first time, observed an unequivocal existence of organ specific properties of the endothelium confirming the structural and functional EC heterogeneity.

Developmentally, endothelium arises from mesoderm via the differentiation of hemangioblasts and/or angioblasts. However, other cell lineages may transdifferentiate into ECs and ECs into other lineages [7]. Precursor of ECs is thought to arise from the ventral floor of the dorsal aorta within the aorta-gonad-mesonephros region. Splanchnopleuric mesoderm transforms into mesenchymal cells, which differentiate into hemangioblasts. The hemangioblasts, then, become pre-ECs, which can further differentiate into either a committed haemopoietic cell line or in an EC [8].

This review will summarize and update the morphological and functional features of the endothelium and it will provide an overview of the major mechanisms participating in the alteration of endothelial functions at cardiovascular level in physiopathological states.

## 2. An Anatomical Overview

The anatomical structure of the endothelium is extremely simple and linear: a single layer of mesenchymal cells; despite the fact that the endothelium is an extremely complex tissue from the metabolic point of view. The EC surface in an adult human is composed approximately of 1 to 6 × 10^13^ cells, weighs approximately 1 kg, and covers a surface area of about 1 to 7 m^2^ [9, 10]. Moreover, the ECs are generally flat, but most of the thickness of the endothelium is determined by a dynamic structure lying on its luminal surface [1]. ECs, that are typically flat, could be also plump or cuboidal in high endothelial venules (HEV) [11, 12] and EC thickness varies from less than 0.1 *μ*m in capillaries and veins to 1 *μ*m in the aorta [3, 13].

Endothelium may be continuous or discontinuous: continuous endothelium, in turn, may be fenestrated or nonfenestrated. The fenestrae are transcellular pores of about 70 nm in diameter extending through the full thickness of the cell and possessing a thin 5 to 6 nm nonmembranous diaphragm across their opening. The density of fenestrae varies through vascular beds [3]. In particular, continuous endothelium is characterized by ECs tightly connected to one another and surrounded by a continuous basement membrane; the subset of the continuous endothelium, defined fenestrate endothelium, has ECs permeated with holes or fenestrae, whereas the discontinuous endothelium is characterized by the presence of fenestrae, frank gaps, and a poorly formed underlying basement membrane [3]. Nonfenestrated continuous endothelium is found in arteries, veins, and capillaries of the brain, skin, heart, and lung. Fenestrated continuous endothelium, however, is characteristic of zone of high filtration or of transendothelial transport, such as capillaries of exocrine and endocrine glands, gastric and intestinal mucosa, choroids plexus, glomeruli, and renal tubules. Discontinuous endothelium is found in certain sinusoidal vascular beds, prevalently at liver level (liver sinusoidal ECs possess larger fenestrations of 100 to 200 nm in diameter, but liver showed a spatial heterogeneity) [3, 14].

Arteries and veins are both lined by continuous nonfenestrated endothelium, but endothelial junctions in arteries are tighter compared with those in veins [15]. Capillaries are characterized by flat ECs surrounded by occasional pericytes and extracellular matrix [5]. Some postcapillary venules are characterised by endothelial-lined bicuspid microscopic valves [16]. These are identical in structure, location, and orientation to the cells of the larger veins valves, with the exception that their leaflets lack fibroblasts and myofibroblasts. The ECs of postcapillary venules are rich in vesiculovacuolar organelles, particularly in the thicker portions of the blood vessels [5, 17]. Postcapillary venules are the preferred site for leukocyte and platelet trafficking, roll, and infiltration inducing endothelial activation in states of inflammation [18, 19], hypercholesterolemia, haemorrhage shock, and ischemia/reperfusion [5, 20, 21].

An important example of EC structural heterogeneity is found in the HEVs of secondary lymphoid organs. Anatomically, HEVs are postcapillary venules, but they are distinct from ordinary venules in several aspects [12] and also ECs in HEVs demonstrate unique structural, molecular, and functional properties compared with other ECs in the body. Each HEV is composed of a prominent perivascular sheath, a thick basal lamina, and a layer of ECs that have a plump morphology [22] and express site-specific adhesion molecules and chemokines that promote the trafficking of lymphocytes between blood and lymph node [5, 12, 23]. Some adhesion molecules are common in both normal venules and HEVs, such as platelet/endothelial cell adhesion molecule-1 (PECAM-1) (also known as CD31), whereas certain molecules are expressed uniquely in HEVs. In particular, peripheral lymph nodes HEVs abundantly express highly glycosylated and sulphated forms of sialomucins, including glycosylation-dependent cell-adhesion molecule 1, CD34, podocalyxin, endoglycan, and endomucin. With respect to normal venules, numerous sialomucins are expressed also in HEVs, but in a glycosylated form. HEVs express also high levels of lymphoid chemokines, although normal venules generally do not, with some exceptions, such as intestine and skin venules [12]. At the ultrastructural level, HEVs are characterized by a prominent Golgi complex, abundant polyribosomes, and rough endoplasmic reticulum, indicating an intense biosynthetic activity not observed in flat ECs. They also contain many membrane-bound vesicular structures, multivesicular bodies, Weibel-Palade bodies, and a variety of dense bodies, indicating that HEVs are involved in secretion. Actually, very few HEV-specific markers are known: the best marker currently available is MECA-79 [11], which stains all HEVs within lymphoid tissues and does not react with postcapillary venules or large vessels in spleen, thymus, or nonlymphoid tissues [24]. HEVs showed also “spot-welded” junctions between high ECs [11, 25] that differ from the tight junctions characteristic of capillary and arterial endothelium, but are similar to the “nonoccluding” junctions found in normal postcapillary venules. These junctions probably facilitate the passage of lymphocytes between adjacent high ECs allowing massive lymphocyte emigration in HEVs [11] (Table 1).

It is important to remember that also heart contains several endothelial compartments, including ECs of coronary arteries, capillaries, and endocardium. These subtypes of ECs differ in developmental origin, structure, and function. Endocardial ECs are larger than other types of ECs and possess many microvilli, which project into the heart cavity [5]. The relative abundance of gap junctions, such as connexins, expressed by endocardium but not by EC of myocardial capillary is due to the fact that the endocardium has more intercellular clefts, gap junctions, and few vesicles [5, 26]. The ECs of coronary arteries are similar in structure and function to ECs of other arteries in the body; whereas capillaries in the heart possess a continuous endothelium and the distance between the capillary EC and the nearest cardiomyocyte is about 1 *μ*m leading to an optimal diffusion of oxygen and nutrients between blood and underlying cells. Like the endocardium, ECs of myocardial capillary are involved in reciprocal signalling with cardiomyocytes [5]. Moreover, cardiac microvascular ECs have been shown to promote cardiomyocyte survival [27]. In the endocardium the expression of von Willebrand factor and endothelial nitric oxide synthase (eNOS) is higher with respect to myocardial microvessels [28, 29] and eNOS is greatly concentrated in the Golgi body, whereas in myocardial capillary ECs, eNOS is more diffusely distributed in the cytoplasm [5, 30].

At cardiac valve level, there are two most prominent cellular components: the valve endothelial cells (VECs) and the interstitial cells. The VECs reside along the entire surface of the cusps or leaflets and are in continuity with endocardium, aorta, and pulmonary artery. The valve interstitial cells, however, make up the collection of mesenchymal cells that reside within specialized extracellular matrix subcompartments, fibrosa, spongiosa, and ventricularis/atrials layers. An extensive study of human VECs, on specimens ranging from 3 weeks gestation to 10 years of age, showed their progressive elongation and flattening on the ventricular side of the cusps, whereas VECs along the arterial side appeared cuboidal. ECs lining cardiac valves exhibit many of the same properties of ECs [31].

## 3. Morphological and Structural Features of Endothelium

The membrane of ECs is made of a double layer of phospholipids separated by water compartments and crossed by complex proteins that work as receptors or channels. ECs possess various contractile proteins, such as actin, myosin, and tropomyosin, that allow motor activities [32]. Some of these proteins are organized in a cortical web that surrounds the internal surface of the sarcolemma and defined ECs shape and elasticity. The junctions associated to actin filament, defined FAU system, are at the intercellular level and their contraction and relaxation control the dimension and hence the shape of the intercellular space, modulating the passage of solutes and macromolecules between blood and subendothelial space. The FAU system is closely related to the intercellular adhesion molecules, especially with vascular endothelial (VE) cadherin, maintaining a balance between adhesive and contractile forces [32].

ECs have three surfaces: cohesive, adhesive, and luminal. The cohesive surface adjoins ECs with each other and facilitates transport processes and consists of specialized intercellular junctions: gap, tight, or adherent junctions and syndesmos [33, 34]. The adhesive surface of ECs adheres to basal lamina and, finally, the luminal side of the vascular endothelium consists of molecules and specific binding proteins regulating trafficking of circulating blood cells [34].

ECs and their nuclei are aligned in the direction of blood flow in straight segments of arteries, but not at branch points [3, 35, 36]. When blood flow increases and so also shear stress, the ECs are flattened and aligned in the direction of the flow; whereas when blood flow decreases the ECs increase their volume losing the alignment and looking like cobble stones paving [32]. The ECs are, in fact, sensitive to changes of the intravascular tension and may increase their stiffness after the increment of the intravascular pressure.

**Figure 1:**
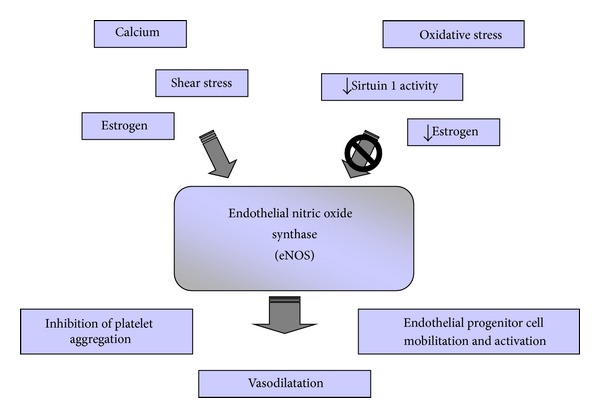
The activation of endothelial nitric oxide synthase (eNOS) may be induced by shear stress, estrogens, or increase of intracellular Ca^2+^ concentration and may be inhibited, mainly during aging physiopathological process, by oxidative stress and reduction of Sirtuin 1 activity and of estrogens level. In healthy endothelium, eNOS plays multiple functions, such as nitric-oxide vasodilation and inhibition of platelet aggregation and may induce endothelial progenitor cells activation and functions.

Bevilacqua et al. [37] identified the first inducible endothelial cell-specific leukocyte adhesion molecule (ELAM-1), later designated as endothelial cell-selectin (E-selectin) [7, 38]. Considering the potential ECs markers, PECAM-1 is, in addition, expressed in monocytes; thrombomodulin as well as in ECs, is also expressed in keratinocytes, trophoblasts and leukocytes and VE-cadherin is expressed also in trophoblasts and fetal stem cells. Moreover, it is known that ECs may express also vascular endothelial growth factor (VEGF) that plays a key role in the generation and maintenance of endothelial fenestrae [3] and eNOS, whose expression increases after higher shear stress, inducing the parallel sustained increase in the production of nitric oxide (NO) [1, 39]. This free radical is the key endothelium-derived relaxing factor that plays a pivotal role in the maintenance of vascular tone and reactivity. In addition to being the main determinant of basal vascular smooth muscle tone, NO acts to negate the actions of potent endothelium-derived contracting factors, such as angiotensin II and endothelin-1 (ET-1). In addition, NO serves to inhibit platelet and white cell activation and to maintain the vascular smooth muscle cells in a nonproliferative state (Figure 1) [40].

Endothelium is also the source of the potent vasoconstrictor peptide ET-1. First isolated, purified, and sequenced in 1988 [41], in health, the production of ET-1 is minimal and it is effectively opposed by NO and other endothelium derived vasodilators [42]. The circulating level of this short peptide was quickly determinant in humans and it was reported that, in most cardiovascular diseases, circulating level of ET-1 was increased [43]. Our research group observed a significative increase of ET-1 expression at aortic level in different animal models: rats with a severe nephrotoxicity induced by cyclosporine A [44, 45], rats with vascular damage-induced by nicotine [46], and in atherosclerotic mice (ApoE-deficient mice) [47]. Molecular data and *in vitro* and *in vivo* findings [48–50] have indicated that angiotensin II can turn on transcription of the precursor of ET-1 gene and biosynthesis of ET-1 in different cell types, including cultured vascular smooth muscle cells [51, 52] and ECs by acting on angiotensin II type 1 receptors. ET-1 might be also involved in the cardiovascular damage because it was found to contribute to hypertrophic response to angiotensin II [50, 53, 54].

Platelet-selectin (P-selectin) is expressed not only in ECs but also in megakaryocytes; it is stored intracellularly in Weibel-Palade bodies and it is expressed preferentially in postcapillary venules [3, 55]. Unlike E-selectin and P-selectin, intercellular adhesion molecule-1 (ICAM-1) and vascular cell adhesion molecule-1 (VCAM-1) are expressed in many vascular and nonvascular cell types. Constitutive expression of cell surface VCAM-1 in mice is generally lower than that of ICAM-1, with the exception of heart, where both are equally expressed, and brain in which VCAM-1 density is 4-fold higher with respect to ICAM-1 [3]. It is well known that the expression of ICAM-1 and VCAM-1 is strictly connected and correlated to atherogenesis [2, 3, 56].

The superoxide dismutase (SOD) enzymes are also a key determinant of NO bioavailability since SOD competes with NO for superoxide in a diffusion-limited manner, thus attenuating the formation of peroxynitrite and, indirectly, improving NO bioavailability [57, 58]. Indeed, in mice with a genetic ablation of the cytosolic isoform SOD1, endothelial dysfunction was associated with increased superoxide and peroxynitrite levels compared with wild type controls. Furthermore, exogenously added SOD was able to partially restore endothelium-dependent vasodilation in an eNOS-dependent manner [58, 59]. In addition, overexpressing the mitochondrial isoform SOD2, specifically in the endothelium of streptozotocin-induced diabetic mice, prevented diabetic retinopathy and superoxide-mediated oxidative stress [60]. These data clearly demonstrate the important role of SOD that plays a role in balancing oxidative stress through removal of superoxide and thereby maintaining NO levels [58].

Endothelial expression of tissue factor has been reported in certain pathologic conditions, including tumors [61], atherosclerosis [62], sickle cell anemia [63], sepsis, and cardiac allograft rejection [64]. It is possible that under these conditions endothelial tissue factor plays an important pathogenic role and that an understanding of its transcriptional regulation would provide new insights into mechanisms of disease [2].

### 3.1. Cellular Junctions

Two main types of intercellular junctions are recognized in ECs: tight junctions, defined also as zona occludens, and adherens junctions, also termed as zona adherens [3, 65, 66]. The junctional composition of intercellular clefts varies across the vascular tree. The tight junctions, which are usually found at the apical region of the intercellular clef, impart two functions in the cell: a barrier function, regulating the permeability of solutes between adjacent cells, and a function controlling the lateral diffusion of proteins within the lipid bilayer [67–69]. The ECs of large artery display a well-developed system of tight junctions due to their conduit function and their exposure to high rates of pulsatile blood flow, whereas in the arterioles the junctions are tighter with respect to capillaries and are quite loose in venules [3]. The extracellular membrane-bound components of the tight junctions are formed by proteins from three families: claudins, occludins, and junction adhesion molecules [70–73].

The identification of several components of adherens junctions in ECs helps in the understanding of the complex role of these structures not only in maintaining cell-to-cell adhesion but also in transferring intracellular signals. VE-cadherin is an endothelial-specific adhesion protein at adherens junctions that interacts with several signalling partners inducing contact inhibition of growth and decrease in permeability.

Several pathological conditions have been associated with altered junction organization including chronic inflammation, atherosclerosis, or tumor angiogenesis. In these cases, altered junctions are likely the consequence of vascular injury or EC activation and retraction [74].

### 3.2. Caveolae

In physiological condition, there are various ways of transporting plasmatic molecules through the endothelial barrier: (i) intercellular unions that generally act as filters controlled by the hydrostatic pressure that allow the passage of water and dissolved substances; (ii) vesicles formed from the caveolae that help the passage of macromolecules through the cell membrane and cytoplasm; (iii) transcellular channels usually formed from various caveolae that connect opposite sides of the cell membrane. In particular, through caveolae, the endothelium regulates the passage of fluid and macromolecules between the vascular and cellular compartments; when this way fails, there is the formation of edema [32].

Caveolae are 70 nm membrane-bound, flask-shaped vesicles that are usually open to the luminal or abluminal side and are occasionally free in the cytoplasm [3]. Caveolae occupy between 5% and 10% of the total EC surface [32, 75]. The number of caveolae is the highest in continuous nonfenestrated endothelium, particularly at heart, lung, and skeletal muscle levels [3], whereas in the blood brain barrier the caveolae are rarely found [15]. It is important to remember that caveolae are present also in non-ECs.

There are three caveolin (cav) isoforms, cav-1, cav-2, and cav-3. Cav-1 and cav-2 are ubiquitously expressed, whereas cav-3 is specific for striated muscle. Cav-1 and cav-3 have a conserved cav scaffolding domain, which is bound by many membrane proteins, such as G proteins, tyrosine kinase receptors, and eNOS [76, 77]. Cav-1 and cav-2 are expressed in most cell types including all cell types of the cardiovascular system, while cav-3 is expressed prevalently in cardiac and skeletal muscle [78].

Cav-1 is the main caveolae found at the endothelial level [58]; various EC signalling molecules localize in caveolae and are modulated by direct interaction with cav-1 [58, 79]. NO production, by eNOS, is tightly regulated by enzyme interaction with cav-1 [80, 81]. Cav-1 has been shown to directly bind eNOS and inhibit eNOS-derived NO release under physiological conditions [82]. On the contrary, for optimal NO release, eNOS must reside in caveolae microdomains [58]. Interestingly, also members of the transient receptor potential channel (TRPC) family reside in caveolae [81, 83, 84].

Our previous study demonstrated that the genetic deletion of cav-1 in mice results in total absence of endothelium-derived hyperpolarizing factors- (EDHF-) mediated vasorelaxation altering calcium (Ca^2+^) entry and disregulating the expression and caveolar location of connexins and myoendothelial and vascular homocellular gap junction components [81]. EDHFs, including NO, carbon monoxide, hydrogen sulphide, lipoxygenase, and others, regulate cellular hyperpolarization decreasing Ca^2+^ influx, either by reducing the open probability of cave channels or the cav channel-dependent activation of the sarcoplasmic reticulum, which induces relaxation of vascular smooth muscle cells [85, 86].

Cav-2 is expressed in ECs, smooth muscle cells, skeletal myoblasts, fibroblasts, white adipocytes, lung tissue, and pancreatic islets. When compared to human cav-1, cav-2 was determined to be roughly 38% identical and 58% similar to a conserved region of eight identical aminoacids. It is important to underline that cav-2 is able to directly bind cholesterol without cav-1 interaction [87].

However, cav-3 is roughly 64% identical to cav-1 and can form homooligomeric complexes with itself and does not require cav-1 to drive caveolae formation [88]. In cardiomyocytes, eNOS localizes to caveolae binding to cav-3. The colocalization of cav-3 and eNOS may facilitate both eNOS activation by cell surface receptors and NO release at the cell surface for intercellular signaling [89, 90].

### 3.3. Transient Receptor Potential Channel

**Figure 2:**
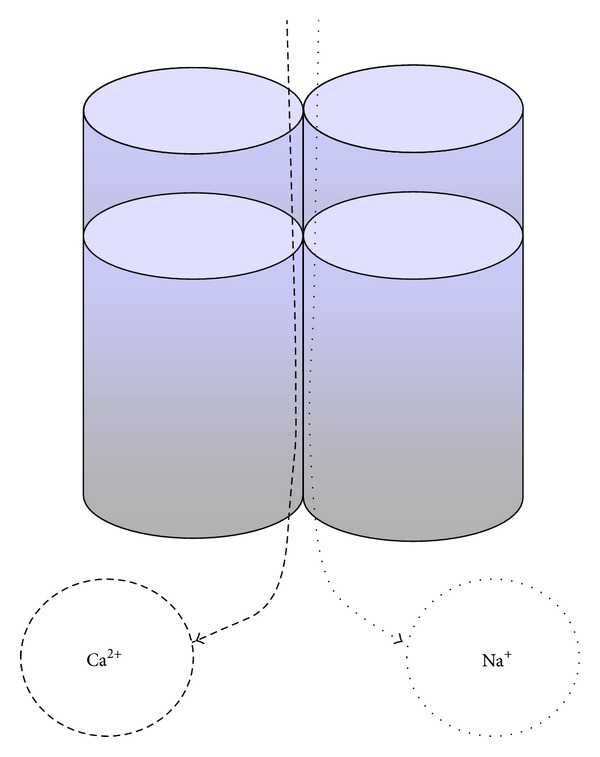
Transient receptor potential channels (TRPCs) clustered around a central ion pore enabling influx of calcium and sodium ions. Ca^2+^: calcium; Na^+^: sodium.

There are six TRPC proteins in humans, but more TRPCs may arise through heteromerization among TRPCs and other types of transient receptor protein. TRPCs support endothelial functions like vascular regeneration, increased permeability, and endothelium-derived NO-mediated vasorelaxation playing a central role in the control of vascular smooth muscle cell tone, endothelial permeability, and platelet function. Some of these ion channels are relatively Ca^2+^ selective but many are nonselective cationic channels with permeability to Ca^2+^, sodium (Na^+^), and potassium (K^+^). In a few instances they are Ca^2+^-impermeable or permeable also to magnesium and other cations (Figure 2). All of the TRPCs are reported to be expressed in blood vessels, especially in ECs and vascular smooth muscle cells. They are functionally important, but almost not critical for the development or maintenance of a physiological vasculature [91].

A careful comparison of EC responses from different TRPC-deficient mice with respect to wild type mice permit to identify which channel(s) is/are essential for modulating the changes in vascular permeability [92].

Freichel and colleagues [93] observed, in primary aortic ECs of TRPC4^−/−^ mice, that TRPC4 is part of the Ca^2+^ influx signal transduction pathway regulating vascular tone. However, TRPC5 might also be involved in this process; in fact, the treatment of bovine aortic ECs with siRNA against TRPC5 prevented NO-induced Ca^2+^ entry and so decreased endothelium-dependent NO vasorelaxation [94]. A TRPC5 and 6 activation cascade have been shown to take part in the regulation of ECs migration [92, 95]. However, inhibition of TRPC3 activity by protein kinase G-dependent phosphorylation was observed to protect ECs from the detrimental effect of excessive NO and Ca^2+^ [92, 96, 97]. In particular, TRPC3 channels have been suggested to serve as redox sensors which monitor oxidative stress in ECs. However, TRPC3 may not be acting alone in this process; in fact the heteromeric channels composed of TRPC3 and 4 form redox-sensitive channels both in native ECs and when being heterologously expressed in human embryonic kidney 293 (HEK293) cells [98, 99].

Nevertheless, the exact role of these channels is still under study. TRPCs may therefore be attractive drug targets to tackle physiopathological states; therapeutic approaches to modulate activation of specific TRPCs are likely to have an important impact in reducing tissue damage in a number of diseases resulting from oxidant stress including ischemia/reperfusion injury, hypertension, edema, bleeding disorders, and diabetes [92, 99].

## 4. Endothelial Progenitor Cells

Endothelial progenitor cells (EPCs) are bone marrow-derived stem cells that differentiate into functional ECs [100–103]. EPCs are mobilised from the bone marrow into the peripheral blood in response to tissue ischemia or injury [104]. EPCs migrate to vascular or tissue injury sites, contributing significantly to reendothelialization and neovascularization and hence tissue repair [105–108] and differentiate into ECs [109]. However, a reduction in the number of circulating EPCs may also result in the impaired reendothelialization of eroded atherosclerotic plaque and thus lead to a greater propensity for thrombosis and vascular occlusion. Several lines of evidence indicate that EPCs constitute an important endogenous system that maintains endothelial and vascular integrity [103].

Moreover, EPCs may play an important role in maintaining an intact and functional endothelium in mature blood vessels and the alteration of this function leads to abnormal vasoreactivity [101]. It was observed by Vasa and colleagues [110] that the number and migratory activity of circulating EPCs decreased in patients with risk factors for coronary artery disease, suggesting that a decrease in the EPCs level may contribute to impaired vascularization. EPCs have been also implicated in postischemic neoangiogenesis [111, 112] and are ideal candidates for vascular regeneration [113, 114]. De Ciuceis and colleagues [115] observed that EPCs and capillary density were reduced in obese subjects, but pronounced weight loss induced by bariatric surgery significantly increased EPCs and seems to induce an almost complete regression of microvascular fibrosis, while not improving capillary rarefaction. Further clinical studies are needed to amplify the knowledge about EPC mobilization and function and for the establishment of EPC measurements as prognostic markers in cardiovascular diseases.

## 5. Endothelial Cells Functions

The endothelium exerts its function in maintaining vascular homeostasis through the balanced release of a number of autocrine and paracrine substances in response to physical, biological, and chemical stimuli [1]. In fact, it is known that the endothelium plays an important role in many physiological functions, including the control of vascular tone, blood cell trafficking, innate and adaptive immunity, and hemostasis [3]. Endothelium is capable of producing vasoactive factors, such as vasodilators and vasoconstrictors, procoagulants and anticoagulants, inflammatory and anti-inflammatory factors, fibrinolytics and antifibrinolytics, oxidizing and antioxidizing, and other factors [32, 116, 117].

The endothelium regulates vascular tone via responding to a variety of stimuli [118]. This process involves a complex interplay between intracellular receptors, the synthesis, and then the release of a variety of endothelium-derived relaxing and constricting substances [119, 120].

Passage of leukocytes from blood to underlying tissue through the endothelial layer involves a multistep adhesion pathway that includes rolling, initial attachment, arrest, and transmigration. These steps take place prevalently in postcapillary venules. In particular, rolling is mediated primarily by interactions between leukocyte carbohydrate-based ligands and endothelial E- and P-selectin and firm adhesion by interactions between leukocyte integrins and ICAM-1 and VCAM-1. There are two pathways for the passage of leukocytes through the endothelial layer: they may pass between ECs, paracellular route, or they may pass through the EC itself, defined as transcellular route [17, 121, 122]. The molecular basis of transmigration is actually controversial, but it is known that it involves CD99, PECAM-1, and junctional adhesion molecule-1 [3, 123, 124].

Another common function of the endothelium is the capacity to maintain blood in a fluid state limiting clot formation when there is a breech in the integrity of the vascular wall and the endothelium serves as a borderline between the coagulation factors circulating in the blood and the primary initiator of coagulation within the vascular wall [86, 125, 126].

ECs express tissue factor pathway inhibitor, heparan, thrombomodulin, endothelial protein C receptor (EPCR), tissue-type plasminogen activator, ecto-ADPase, prostacyclin, and NO, as anti-coagulant factors, whereas ECs may synthesize tissue factor, plasminogen activator inhibitor-1, von Willebrand factor, and protease activated receptors (PARs), as procoagulant factors [3, 127]. EPCR is expressed predominantly in large arteries and veins [128], whereas thrombomodulin is highly expressed in blood vessel types of every calibre in all organs, with the exception of the brain, where its expression is low [3, 129]. The differential distribution of procoagulants and anticoagulants in the vessel tree suggests and confirms the endothelial heterogeneity and that ECs from different sites of the body use site-specific procoagulants or anticoagulant factors to balance specific and local haemostasis [7].

**Figure 3:**
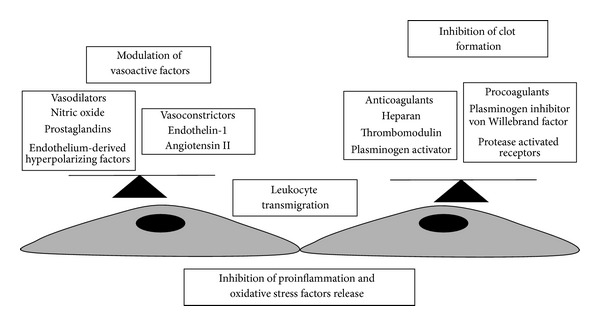
Endothelial functions in physiological conditions. The net balance of endothelium-derived vasodilators and vasoconstrictors or anticoagulants and procoagulants along with inhibition of proinflammatory and oxidative stress factors release and leukocyte adhesion and transmigration maintain a healthy vascular homeostasis.

Other important functions of the endothelium are to regulate the transport of liquids across the semipermeable vascular endothelial barrier [92, 125] and function as a protective biocompatible barrier between all tissues and the circulating blood, hence modulating the bidirectional passage of macromolecules and blood gases to and from tissues and blood [41]. Importantly, these properties vary both in space and time, once and again giving rise to the phenomenon of EC heterogeneity [2, 130]. In Figure 3 the main functions of endothelium are summarized.

Given the critical role of these mechanisms in which ECs are the key factors, the deregulation of the endothelial balance, defined endothelial dysfunction, leads to the pathogenesis of many diseases including atherosclerosis, hypertension, sepsis, and some inflammatory syndromes [1, 40].

## 6. Endothelial Dysfunction

As described in the previous paragraphs, the endothelium is an emergent and complex system. It is multifunctional, highly distributed in space, and has an enormous behavioural repertoire; in fact EC dysfunction is not restricted anatomically to a single organ or limited in a singular disease mechanism [3, 7]. The ECs represent a powerful organizing system in human health and disease, also because they are involved in numerous pathological states either as primary determinants of physiopathology or as victims of collateral damages [5, 7].

Endothelial dysfunction disrupts the mechanism of vascular homeostasis regulation predisposing the vessel wall to vasoconstriction, leukocyte adhesion, platelet activation, oxidative stress, thrombosis, coagulation, and inflammation hence leading to the pathogenesis of cardiovascular diseases [40, 131].

Early description of EC dysfunction focused on structural changes or loss of anatomical integrity, particularly in the context of atherosclerosis: Ross and Glomset [132] proposed, in 1973, a response-to-injury hypothesis to explain the lesions of atherosclerosis. Then, Bevilacqua et al. [37] employed the term EC dysfunction to describe hyperadhesiveness of the endothelium to platelets.

It is important to underline that EC may be activated without being dysfunctional [7] and that endothelium is highly active and constantly sensing and responding to alterations of the local extracellular environment [133]. The term “activation” reflects the capacity of ECs to perform new functions without evidence of cell injury or dysfunction [134].

It is well known that the intact endothelium may actively contribute to disease initiation and/or progression. The transition between EC function and dysfunction is not always clear. EC dysfunction usually arises from otherwise adaptive responses that are now excessive, sustained, or spatially and/or temporally misplaced [7]. Moreover, the endothelium is heterogeneous in its response to physiopathological stimuli [133].

The concept of EC activation first arose from *in vitro* studies demonstrating the ability of well-defined stimuli to induce the expression of the so-called “activation antigens” on the surface of ECs; actually, P-selectin is considered a marker of EC activation [135].

In conclusion, the endothelium is highly plastic and thus amenable to therapeutic modulation, in establishing a dialogue with the underlying tissue and so it provides a possible direct line of communication with every organ in the body. The goal in treating the endothelium is not to reset the switch, but rather to fine-tune and recalibrate the EC, nudging back to their physiological state [133]. Given that EC phenotypes vary according to time and location in the vascular tree, in both health and disease states, it is essential to modulate therapy to specific vascular beds.

In the following paragraphs, the main EC dysfunction observed at cardiovascular level in the pathological states of atherosclerosis, hypertension, diabetes, and cardiac valvular degeneration or induced by age or cigarette smoking was summarized. Successively, we will briefly present a possible emerging prevention/treatment of ECs dysfunction through a healthy lifestyle.

### 6.1. Atherosclerosis

Endothelial dysfunction is known to be implicated in the pathogenesis and clinical course of all known cardiovascular diseases [136, 137], occurs in response to cardiovascular risk factors, and precedes the development of atherosclerosis [42, 138, 139]. Endothelial dysfunction actively participates in the process of lesion formation promoting the early and late mechanisms of atherosclerosis [32, 40, 42], determining increase in EC permeability, upregulation of adhesion molecules, chemokine and cytokine secretion, and leukocyte adherence, enhanced oxidized-low density lipoprotein (ox-LDL), platelet activation, and vascular smooth muscle cell proliferation and migration. In addition, endothelial dysfunction is not only the initial stage of the development of atherosclerotic disease that generates plaque formation, but it also can cause plaque growth leading to vascular complications. For all these reasons, endothelial dysfunction is one of the principal mechanisms in atherosclerotic diseases [32].

The endothelial injury, activation, and dysfunction caused by ox-LDLs in the pathogenesis of atherosclerosis are exerted via the activation of lectin-like ox-LDL receptor-1 (LOX-1) activation [100, 140]. LOX-1, initially identified as the major receptor for ox-LDL in ECs, can also be expressed in macrophages and smooth vascular muscle cells [101, 141–143]. LOX-1 has the ability to bind damaged or apoptotic cells, activated platelets, advanced glycation end products, and pathogenic organisms [144, 145] and so it may play a role in initiating and potentiating the early steps of atherogenesis. However, elevated LOX-1 expression is observed not only in both initial and advanced atherosclerotic lesions [101, 143, 146, 147] but also during other cardiovascular injuries, such as hypercholesterolemia, hypertension, obesity, and diabetes [140, 148]. Ox-LDL, via upregulation of LOX-1 mediated by angiotensin II and ET-1, induces monocyte adhesion to the endothelium via enhanced expression of P-selectin, ICAM-1, and VCAM-1 [149, 150]. Receptor activation also results in monocyte chemoattractant protein-1 expression, promoting monocyte migration into the intima [101, 151]. However, ox-LDL uptake by LOX-1 also mediates EC apoptosis, potentially via nuclear factor (NF)-*κ*B activation [101, 152] and resulting in direct vascular denudation and injury that may trigger or enhance the inflammatory and oxidative stress reactions. Ox-LDL binding to LOX-1 induces an increase production of intracellular reactive oxygen species (ROS), apoptosis of vascular smooth muscle cells, and modulation of matrix metalloproteinase activity, all factors/conditions that compromise and alter the atherosclerotic fibrous cap. Moreover, activated platelets may interact with the surrounding endothelium via LOX-1 [153] promoting the release of ET-1 from ECs, stimulating the generation of ROS that inactivates NO [145] and so starting a vicious circle that potentially ends in vascular occlusion and ischemic insult.

ECs are involved in the atherogenic process also through PAR expression. EC PAR-1 activation promotes adhesivity toward monocytes via the induction of NF-*κ*B, which in turn promotes ICAM-1 expression [101, 154]. PAR-1 and PAR-2 activity induces discharge of Weibel-Palade bodies and endothelial storage granules containing the adhesion molecule P-selectin and von Willebrand factor, promoting both leukocyte and platelet adhesion [101, 155]. PAR activation is also linked to the secretion of interleukins and cytokines that promote C-reactive protein synthesis that, itself, triggers many of the steps in the inflammatory process so enhancing the initiation and progression of atherosclerotic plaques [156]. Moreover, atherogenesis progressively impairs endothelial vasoactive function, ranging from impaired endothelium-dependent vasodilatation in conduit and resistance of vessels in patients with hypercholesterolemia to complete loss of endothelium-dependent vasodilation in patients with histologically proven atherosclerotic lesions [157–160].

Lipid-lowering diet [161, 162] and lifestyle modification [163, 164] have been shown to restore or improve impaired endothelium-dependent vasodilation in the atherosclerotic disease state [160].

### 6.2. Hypertension

Arterial hypertension is the most prevalent risk factor associated with increased cardiovascular morbidity and mortality [165]. The hypertensive vascular dysfunction, characterized by endothelial dysfunction and hypertensive remodelling of vascular smooth muscle cells, are well known and described processes. The complicated mechanisms underlying vascular dysfunction involve decreased NO bioavailability, activation of the pathways of vascular smooth muscle contraction, vascular oxidative stress, and inflammation [166–169]. The impaired endothelium-dependent vasodilatation in hypertension is characterized by an imbalance between EC derived vasodilatant and vasoconstrictor factors [170–172]. Oxidative stress is important in the development and maintenance of hypertension, in terms of excess production of oxidants, decrease in NO bioavailability, and antioxidant capacity in the vasculature [169, 173]. Moreover, the prevalence of hypertension markedly increases with advancing aging. Although aging and hypertension, either independently or collectively, impair endothelial function, they may have similar cascades for the pathogenesis and development of endothelial dysfunction [174].

At this regard, reductions in both vascular oxidative stress and inflammation have been shown, also by our research group, that reverse endothelial dysfunction through the administration of antioxidants, such as melatonin or pycnogenol, in an experimental model of genetic hypertension [175–177].

### 6.3. Diabetes

Diabetes, characterized by persistent elevation of blood glucose levels (hyperglycaemia), occurs due to inadequate production of insulin (type 1 diabetes) or resistance to endogenous insulin usually associated with metabolic syndrome and obesity (type 2 diabetes).

Endothelial functions impaired in metabolic syndrome could reduce insulin access to the tissue and thus decrease insulin sensitivity independently from direct effects at the muscle cells [178]. In type 1 diabetes, endothelial dysfunction is predominantly triggered by the metabolic changes related to hyperglycemia and microvascular complications, but prevalently at retinal and kidney levels [179]. In type 2 diabetes, the link between endothelial dysfunction and diabetes is more complex, as endothelial dysfunction starts well before the onset of diabetes [180–182].

In insulin resistance and diabetes, a variety of endothelial functions is compromised, including regulation of vascular tone [182, 183] and organ perfusion [184, 185], inhibition of inflammation [182, 186], transendothelial transport of blood solutes [182, 186], prevention of coagulation [178, 185], and initiation of angiogenesis [187, 188].

Moreover, it has been shown that impaired endothelium-dependent vasorelaxation is linked to increased cav-1 protein expression in the aorta of diabetic rats. This was attributed to an inhibition of eNOS function due to cav-1 binding and a reduction of NO production [189, 190].

The endothelial insulin signalling is significantly impaired after a high fat diet, coinciding with a reduction in insulin-induced capillary recruitment and reduced interstitial insulin. Thus, it suggests that the endothelial insulin signalling required for delivery of insulin to the interstitial space can be inhibited physiologically by diet [178].

**Figure 4:**
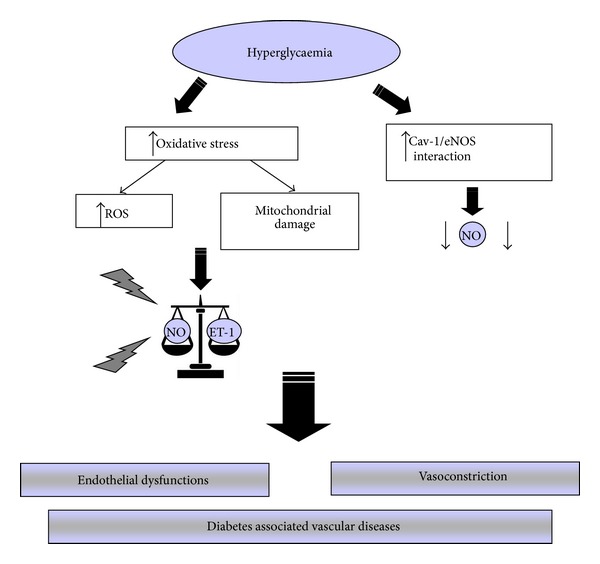
Main pathways activated during hyperglycaemia and low insulin level that alter the modulation of vascular tone, reducing nitric oxide, and increasing endothelin-1 release and so lead to endothelial dysfunction and diabetes-associated vascular disease. Cav-1: caveolin-1, eNOS: endothelial nitric oxide synthase; ET-1: endothelin-1; NO: nitric oxide.

Recent studies have observed that hyperglycemia caused mitochondrial fragmentation and altered mitochondrial dynamics, associated with increase in mitochondrial ROS production [191] and so could cause a rapid breakdown in NO vascular tone regulation [58, 178]. This impairment could be responsible for the endothelial dysfunction observed in diabetes; however, endothelial dysfunction is often evident prior to a significant elevation in plasma glucose levels and can be induced by factors other than hyperglycaemia [178, 192]. In Figure 4 are schematically summarized the main pathways activated during hyperglycaemia and low insulin level inducing diabetes-associated vascular diseases.

Adipose tissue, especially in the abdomen and around blood vessels, defined perivascular adipose tissue (PVAT) [182, 193], has been shown to control insulin sensitivity and endothelial function [194], especially insulin-mediated vasoreactivity [182, 195–199]. Adipose tissue secretes a wide variety of bioactive substances (adipokines) that act directly on vascular endothelium [200]. With regard to endothelial function, PVAT is increasingly recognized as a critical fat depot that regulates local vascular tone [168] and inflammation [182, 201, 202]. These functions appear impaired in obesity and type 2 diabetes [168, 184, 203]. The PVAT inflammation may not only contribute to endothelial dysfunction but also hypoperfusion of adipose tissue and the resulting hypoxia may also trigger inflammation and alter adipokine secretion hence leading to a dysfunctional modulation of vascular tone [182, 204, 205].

### 6.4. Cardiac Valvular Degeneration

Cardiac valve pathology may be associated with local expression of VCAM-1 and E-selectin [5, 206, 207]. There is also evidence that aortic valve stenosis is associated with angiogenic activation of valvular ECs. In Chalajour et al.'s [208] study, normal valves were avascular, whereas stenotic aortic valves contained neovessels. ECs lining these neovessels were consistently positive for PECAM-1, but only a portion was positive for von Willebrand factor. The heart valve stimulated by myocardium signals may lead to ECs transformation in endocardial-mesenchymal cells, losing cell-cell contacts and invading the extracellular matrix so forming endocardial cushions. It is important to underline that only those ECs within this region are capable of responding to these signals [5].

Halcox and Quyyumi [209] showed that impaired endothelium-dependent vasodilatation in coronary arteries with established atherosclerosis induced vasoconstriction, which may result in reduced myocardial perfusion and myocardial ischemia. There are also evidences to suggest that abnormalities of endothelium-dependent vasodilatation contribute to the clinical syndromes of microvascular angina and coronary vasospasm [42].

### 6.5. Cigarette Smoking and Endothelial Dysfunction

**Figure 5:**
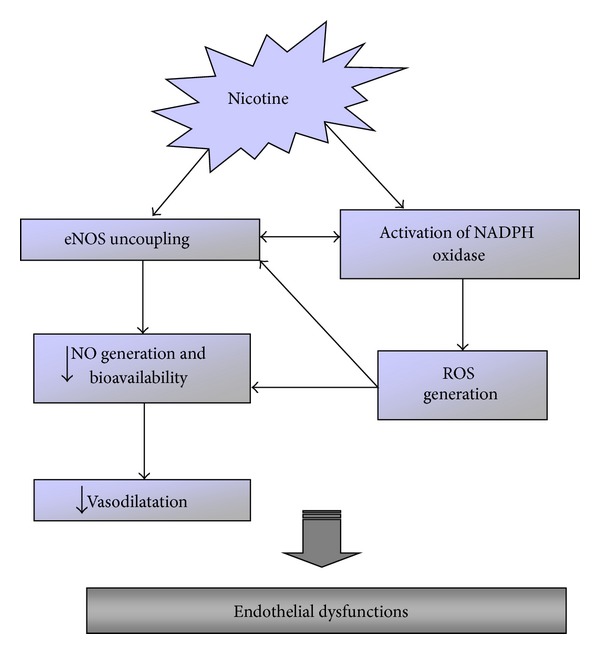
Schematic representation of endothelial dysfunction induced by chronic exposure to nicotine.

Epidemiological studies suggest that cardiovascular diseases account for over one-third of deaths of cigarette smokers [210, 211]. Nicotine exposure via chronic cigarette smoking is an emerging cause of cardiovascular disorders [212, 213]. Numerous studies suggest that exposure to cigarette smoke leads not only to EC and vascular smooth muscle morphological alterations, but also functional exchanges [214–216] as early events in the pathogenesis of cardiovascular disease induced by cigarette smoking [217]. Nicotine plays a key role in mediating these changes by decreasing NO generation and bioavailability and downregulating the expression of eNOS (Figure 5) [218].

Furthermore, nicotine causes a loss of functional integrity of endothelium by causing vasospasm, stimulating the adhesion of platelets and leukocytes and promoting the formation of thrombus [219]. Our research group demonstrated that the administration of nicotine in rats depleted the bioavailability of NO, increased ROS, and damaged the structural integrity of aortic endothelium inducing cardiovascular disorders. The possible mechanism proposed to explain the damage of nicotine was the disruption of the physiological balance of vascular tone, the increased expression of ET-1, inducible NOS, and the reduced expression of eNOS and so NO and SOD [220, 221]. Furthermore, we observed both an increase in ICAM-1 and VCAM-1 expression that induced, in turn, the adhesion of monocytes and lymphocytes at EC level promoting the formation of the atherosclerotic lesion [46]. Nicotine may induce also the release of platelets derived growth factors, which promote the migration of vascular smooth muscle cells at the subendothelial space [222].

### 6.6. Aging and Endothelial Dysfunction

Aging is one of the main risk factors for the development of cardiovascular diseases and dysfunction at both endothelial and vascular smooth muscle cells. This dysfunction favours vasospasm, thrombosis, penetration of macrophages, cellular growth, oxidative stress, and inflammation leading to atherosclerosis and it is considered as a crucial event in the development of many vasculopathies [223]. Moreover, the aging process may deteriorate the balance between vasodilator and vasoconstriction substances produced by the endothelium [224–227]. This imbalance is mainly characterized by a progressive reduction of NO bioavailability and an increase in the production of cyclooxygenase-derived vasoconstrictor factors [223–227]. Both circumstances are in turn related to an increased production of ROS and reactive nitrogen species [223, 226]. Free radicals play a physiological role in the vessel wall; in fact, they participate as second messengers in endothelium-dependent functions, vascular smooth muscle, ECs growth and survival, and in remodelling of the vessel wall [227, 228].

The presence of endothelial dysfunction in old people is associated not only with cardiovascular diseases such as atherosclerosis, coronary artery disease, diabetes mellitus, and arterial hypertension [228] but also with diseases related with aging as renal dysfunction [229], Alzheimer's disease [223, 230], circadian cycle alterations [231], erectile dysfunction [232], osteoporosis [223, 233], and retinopathy [234]. In addition, cell senescence plays a key action in the attenuated angiogenic and regenerative capacity of ECs with aging [223]. Senescent ECs showed a reduced proliferation that may limit the capacity to form new vascular structures. Interestingly, initial signs of endothelial senescence can even be found in young people [235, 236].

Several studies showed mitochondrial oxidative stress as a typical age-related endothelial dysfunction. This phenomenon is associated with the overactivation of nicotinamide adenine dinucleotide phosphate (NADPH) oxidase, an enzyme localized in the cytoplasm and in membranes of mitochondria [227, 237, 238]. Other molecular mechanisms responsible for age-related mitochondrial oxidative stress in the vasculature involve deregulation of antioxidant defences, such as peroxynitrite-mediated erythroid 2-related factor 2 (Nrf2) dysfunction, nitration and inhibition of SOD, decline in glutathione content, and a dysfunctional electron transport chain [227, 239]. Previous studies suggest that increased ROS in aging promotes mitochondrial protein oxidation and increased mitochondrial DNA mutations in heart and other organs, but it is yet to be determined whether similar aging-induced mitochondrial DNA and protein damages play an important role in ECs and vascular smooth muscle cells alterations. Furthermore, some evidences suggest that mitochondria-derived ROS contribute to accelerated development of the senescent phenotype in ECs. EC senescence may impair the physiological properties of the endothelium and may promote the progression of cardiovascular diseases by altering the secretion of cytokines, growth factors, and proteases in the vascular wall [240].

Inhibition of Sirtuin 1, a class III nicotinamide adenine dinucleotide- (NAD-) dependent protein deacetylases defined as longevity protein [47, 241, 242], induces premature senescence, whereas Sirtuin 1 overexpression reverts premature senescence induced at vascular level [227].

In our recent study, we observed that Sirtuin 1 is implicated in the development of age-related vasculopathy. Sirtuin 1 is not present in the vessels of apolipoprotein E-deficient mice at 6 weeks or 15 weeks of age. So, the studies provide a new insight into the atheroprotective effects of Sirtuin 1 and imply that Sirtuin 1 may be a potential target for the intervention in the atherogenetic process [47].

**Figure 6:**
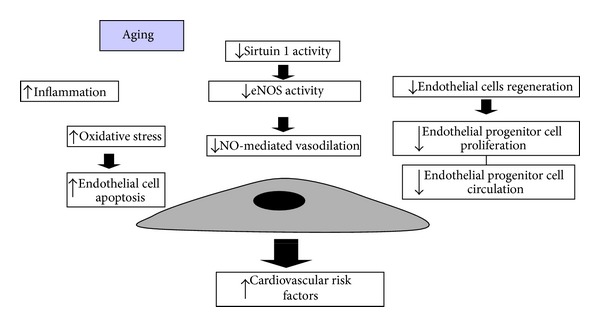
Schematic summary of the main age-related endothelial alterations.

The main EC dysfunction induced by the physiopathological process of aging is summarized in Figure 6. Further studies are warranted to determine whether novel therapies that reduce mitochondrial oxidative stress and/or senescence of ECs are able to prevent the development of endothelial senescence and apoptosis, improve vasodilator functions, and prevent age vasculopathy [240].

## 7. Prevention of Endothelial Dysfunction

Various pharmacological therapies have been designed to reduce the development and progression of cardiovascular diseases, such as insulin sensitizers, statins, Ca^2+^ channel blockers, inhibitors of the rennin-angiotensin system, and antiplatelet agents [243–245]. However, strictly controlling cardiovascular risk factors is often difficult to obtain and the progression of pathological states has not been completely prevented with current pharmacological therapeutic options. Moreover, the modern evolution of Western societies seemingly steers populations towards a profound sedentary lifestyle and incorrect diet is becoming difficult to reverse. Understanding the mechanisms that explain the fatal effects of physical inactivity and incorrect diet opposite to the beneficial effects of a healthy lifestyle remains largely unexplored [243, 246].

In the following paragraphs, we briefly present the beneficial effects of a healthy life style on ECs dysfunction.

### 7.1. Physical Activity

A large body of works has shown that moderate-intensity aerobic exercise training improves endothelial function in animals and humans with and without cardiovascular risk factors [247–251]. The multiple benefits of physical activity on survival and the harmful effects of a sedentary lifestyle made it a therapeutic modality potentially useful to treat patients with cardiovascular diseases. Its benefits regarding functional capacity can be explained by effects on endothelial properties and peripheral vascular resistance [252]. Exercise training reduces inflammation and an improvement in EC health could be a primary reason for the improvement in chronic inflammation [247].

Many studies have demonstrated evidence of abnormal endothelial function among patients with heart failure, which can be mitigated by exercise training [249]. Chronic heart failure is associated with increased levels of tumour necrosis factor-*α* and markers of endothelial damage, including ICAM-1 and E-selectin. Whereas acute bouts of exercise lead to an increase in proinflammatory cytokines and markers of endothelial damage, these effects were not seen when exercise was performed chronically in chronic heart failure patients [250, 251]. Ten weeks of moderate intensity exercise training improved coronary arteriolar endothelial function and reduced tumour necrosis factor-*α* at heart level of type 2 diabetic mice [252]. It is interesting to note that diet plus exercise training may exert remarkable benefits when either diet or exercise alone could not demonstrate so evident benefits [251].

We believe that further investigations in this exciting field would facilitate the development of physical exercise and dietary supplements as adjunctive therapies in the management of cardiovascular diseases.

### 7.2. Diet

Numerous studies have shown that the acute administration of a high fat diet induces a transitory disruption of endothelial function. This effect has been observed with different types of fats, including saturated, monounsaturated (MUFA), and trans fatty acids [243, 253]. It has been estimated that over 2% of total calorie intake in the developed world comes from foods containing trans fatty acids [254]. Fatty acids, produced industrially by partial hydrogenation of vegetable oils, are present in semisolid fats used in the manufacture of margarine, pastry products, frozen foods, and others. Trans fatty acids are proinflammatory factors and induce endothelial dysfunction [255, 256], which may explain the relationship between their consumption and the risk of ischemic heart disease, sudden death, and possibly diabetes [257, 258].

**Figure 7:**
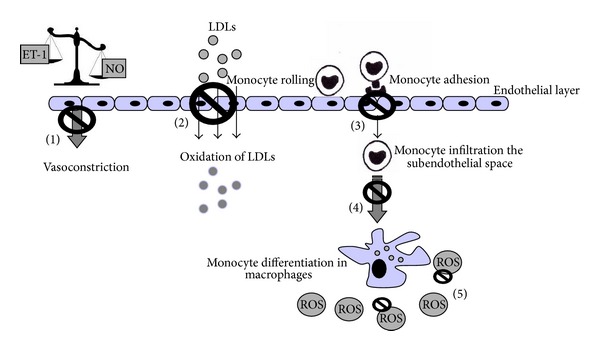
Potential atheroprotective role of Mediterranean diet during the atherogenetic process. The Mediterranean diet, with mainly its antioxidant properties, may (1) inhibit/reduce the imbalance between vasoconstrictors and vasodilators, (2) block oxidation of low density lipoproteins, (3) inhibit monocytes adhesion to endothelial cells constraining monocytes infiltration in the subendothelial space, (4) repress differentiation of macrophages in lipid-laden macrophages, defined as foam cells, and (5) reduce radical oxygen species syntheses. ET-1: endothelin-1; LDLs: low density lipoproteins; NO: nitric oxide; ROS: radical oxygen species.

It has been found that Mediterranean diet, characterized by a low content of saturated fats, reduces endothelial dysfunction and markers of vascular inflammation in patients with the metabolic syndrome [259, 260], as well as reducing insulin resistance [256, 261]. de Lorgeril et al. [262] confirmed that the benefits of the Mediterranean diet, in patients recovering from myocardial infarction, are due not only to its low saturated fat and linoleic acid content, but also to the quantities of vegetables and oleic and *α*-linolenic acid, both found in olive oil. One possible explanation of the effects of unsaturated fatty acids is that they decrease ICAM expression, which would reduce leukocyte adhesion to dysfunctional endothelium [263]. However, in a study on a hundred patients with ischemic heart disease it was shown that a Mediterranean diet for one year did not change inflammatory or metabolic (cholesterol and triglycerides) biomarkers [256, 264]. Ong et al. [265] showed that also a diet rich in MUFA produces impairment in endothelial function when compared to a carbohydrate-rich meal. Similarly, it was observed that a diet rich in olive oil produces the same decline in flow-mediated vasodilatation as did a fast food meal [243, 253]. Lowering saturated fat intake significantly reduced atherosclerosis progression and ICAM-1 levels after two years [256, 263]. Polyunsaturated fatty acids (PUFA) appear to protect the endothelium through various mechanisms: inhibiting expression of VCAM-1 and inducing NO synthesis [256, 266]. However, it is important to remember that slimming diets low in carbohydrates and high in protein [267] or fats [268] or low in fats and high in carbohydrates [269] may reduce weight and improve lipid profile, potentially reducing atherosclerosis and risk for coronary heart disease, but they also have significant drawbacks such as ketosis, nutritional imbalances, significant renal and liver damage, and also atherosclerotic coronary disease and obesity [256, 270, 271]. The potential atheroprotective role of Mediterranean diet during the atherogenetic process is represented in Figure 7.

Several randomized trials have shown a beneficial effect of consuming the Mediterranean diet on markers of endothelial function [258, 272, 273]. A randomized crossover study of Marin and colleagues [274] in 20 Italian elderly people evaluated the effect of a Mediterranean diet on circulating endothelial microparticles and EPCs. Activated endothelial microparticles are complex vesicular structures shed from activated or apoptotic ECs and play a remarkable role in coagulation, inflammation, and angiogenesis contributing to the progression of vascular diseases; in particular activated endothelial microparticles are released in response to the damage of the vascular endothelium, whereas EPCs are involved in the maintenance and replacement of ECs. After 2 weeks, the Mediterranean diet lowered plasma concentrations of total activated and apoptotic EMPs and increased concentrations of EPCs as compared with a high saturated fat or low-fat diet. These results suggest and confirm that the Mediterranean diet may have beneficial effects on endothelial function and so cardiovascular diseases [272].

Ingestion of high levels of saturated fat [273] appear to cause rapid alterations in endothelial function, including reduced vasodilatation. Conversely, the vasodilation induced by a low-fat high carbohydrate diet could be attributable to increased production of NO by the endothelium [256, 275]. A study of Pérez-Jiménez et al. [276] showed that the Mediterranean diet for 28 days to healthy subjects produces a decrease in plasma markers of endothelial activation, suggesting a consequent improvement in endothelial function. Similarly, the chronic consumption of low-fat diets and Mediterranean diets was observed to improve endothelial function compared to a high-fat western diet [243, 277]. Leighton et al. [278] evaluated the effects of two high fat diets: MUFA and PUFA, both of these diets contain exiguous fruits and vegetables and were administered to healthy male during 3 weeks. It was detected that both diets, independent of the type of fat, elicited a significant decline in endothelial-dependent vasodilatation. This negative effect was reverted when the subjects added two glasses of red wine per day or fruit and vegetables to their diet, suggesting that high-fat diets induce EC dysfunction that can be counteracted with the consumption of natural antioxidants so that a dietary pattern rich in fruit, vegetables, fish, and olive oil appears to have beneficial effects on endothelial functions.

#### 7.2.1. Polyphenols

A large number of studies have investigated the role of dietary components such as polyphenols and antioxidants, proposing that diet may prevent endothelial dysfunction hence improving endothelial function [272]. Polyphenols are the most abundant antioxidants in human diet and are widespread constituents of fruits, vegetables, cereals, olive, legumes, chocolate, and beverages, such as tea, coffee, and wine [279, 280]. It is important to underline that many dietary polyphenols possess both direct and indirect antioxidant activities. The term direct and indirect antioxidants was originally used by the Food and Drug Administration to distinguish nutrients that “*trap and deactivate reactive oxygen molecules*” (e.g., vitamin C, vitamin E, *β*-carotene) from those that are “*precursors of coenzymes that are involved in oxidative stress but do not have direct antioxidant activities*” (e.g., zinc, selenium, riboflavin). Later, these terms were redefined by Dinkova-Kostova and Talalay [281] who described two types of small-molecule antioxidants that protect against cellular oxidative damage [282, 283]. Various studies indicate that polyphenols may induce the upregulation of endogenous antioxidant enzymes and cytoprotective proteins such as quinone oxidoreductase-1, SOD, glutathione S-transferase, glutathione peroxidase, and heme oxygenase-1 hence exerting also an indirect antioxidant effect in addition to direct radical scavenging [283, 284].

Despite their wide distribution, the health effects of dietary polyphenols have been attentively studied only in recent years and several studies, although not all, have found an inverse association between polyphenol consumption and cardiovascular disease mortality [285].

Moreover, flavonoids, many of which are polyphenolic compounds, are believed to be beneficial for the prevention and treatment of cardiovascular diseases mainly by decreasing oxidative stress and increasing vasodilatation [279, 286, 287]. More than 8.000 different flavonoids have been described and since they are prerogative of the kingdom of plants, they are part of human diet with a daily total intake amounting to 1 g, which is higher than all other classes of phytochemicals and known dietary antioxidants. In fact, the daily intake of vitamin C, vitamin E, and *β*-carotene from food is estimated minor of 100 mg [279]. A large number of human intervention studies have evaluated the beneficial effects of flavonoid intake and foods containing flavonoids on endothelial function. Morand et al. [288] investigated the acute and 4-week effects of orange juice and its major flavonoid, hesperidin, on microvascular reactivity in a crossover study in 24 healthy overweight men and observed that the intake of orange juice flavonoids may have acute beneficial effects on endothelium but it does not provide evidence for a long-term administration. Moreover, epidemiological studies have suggested that regular moderate consumption of red wine, rich in flavonoids, is associated with increased NO expression hence the positive modulation of vascular tone and that consumption of cacao and green tea improved endothelial functions and this effect appears to be at least partly mediated by flavonoid components, like catechins.

Similarly, another study suggested that short- and long-term black tea consumption reverses endothelial dysfunction in patients with coronary heart disease [243, 289].

For other foods such as citrus fruit and apples, the evidence for beneficial effects on endothelial function is currently under consideration.

The main problem in studing the beneficial effects of foods is judging whether the effect comes from specific compounds or from other bioactive compounds of these foods. Only a few intervention studies have been conducted with pure compounds, so it is important to take into account the possible synergistic effects of multiple dietary components that are part of a dietary pattern and so simulate better the reality of human lifestyle.

In the following paragraphs, we briefly describe the beneficial effects on the endothelium of two of the main food components of the Mediterranean diet, olive oil and red wine, and of some of the principal antioxidant supplementation actually studied [243].

#### 7.2.2. Olive Oil

Olive oil is the main fat in the Mediterranean diet. The wide range of antiatherogenic effects associated with olive oil consumption could contribute to explain the low rate of cardiovascular mortality found in southern European Mediterranean countries, in comparison with other western countries, despite a high prevalence of coronary heart disease factors [290]. Olive oil, particularly a virgin olive oil rich diet, decreases prothrombotic environment, modifies platelet adhesion, coagulation, and fibrinolysis, and may improve endothelial function [290, 291]. The healthful properties of olive oil have been often attributed to its high content of MUFA, namely, oleic acid [292], but it is becoming clear that the content of oleic acid alone cannot fully explain the impact of olive oil on health. It should be underlined that olive oil, unlike other vegetable oils, contains high amounts of several micronutrient constituents, including polyphenolic compounds (100–1000 mg/kg) [293]. Around 80% or more of the olive oil phenolic compounds are lost in the refination process; thus, their content is higher in virgin olive oil than in other olive oils.

The cardioprotective effects of olive oil have been ascribed to its content of MUFA and the presence of other biologically minor constituents like polyphenols, tocopherols, and triterpenoids [260, 294, 295]. Among these compounds, tocopherols and phenolic compounds have demonstrated antioxidant properties that may improve endothelial function by reducing levels of ROS in the endothelium and, consequently, the production of endothelial adhesion molecules. Moreover, extra virgin olive oil through its phenolic compounds may increase glutathione reductase and glutathione peroxidase activities [296, 297].

At this regard, some findings suggest that both major and minor olive oil components may modulate inflammation and endothelial activation. In cultured ECs, oleic acid inhibited the expression of VCAM-1 mRNA levels, monocyte adhesion, and NF-*κ*B [298, 299]. In animal models, a diet rich in olive oil suppressed natural killer cell activity and the expression of receptors for interleukins [300, 301]. Isolated human LDL enriched in oleic acid reduced monocyte chemotaxis and adhesion, compared with linoleic-enriched LDL when being exposed to oxidative stress [290, 302]. Phytosterols and triterpenoids have anti-inflammatory and vasodilatation effects, respectively, but their roles in the endothelium need to be further studied. The increasing investigations on the properties of these minor compounds of olive oil may help to explain not only some of the classic beneficial effects of the Mediterranean diet, but also the emergence of other olive-derived oils, such as pomace olive oil, which, being enriched with these minor components, might be helpful in preventing cardiovascular diseases [291].

Olive oil rich Mediterranean diet has been observed to improve the endothelium-dependent dilatation in hypercholesterolemic males [277, 290] and metabolic syndrome patients [261]. Ryan et al. [261] showed that an olive oil diet attenuates the endothelial dysfunction present during the consumption of a baseline diet high in PUFA. Moreover, Ruano et al. [303] reported that a meal containing high-phenolic virgin olive oil improves the endothelial-dependent vasodilatation during postprandial state more than when the meal was taken with a similar olive oil, but with low-phenolic content.

Olive oil minor components have also been involved in the antioxidant activity of olive oil. Some components of the unsaponifiable fraction, such as squalene, *β*-sitosterol or triterpenes, have been shown to display antioxidant and chemopreventive activities and capacity to improve endothelial function decreasing the expression of cell adhesion molecules and increasing vasorelaxation [304].

The mechanisms by which olive oil and its components exert beneficial effects merit further investigation and further studies are required to obtain evidence of the benefits of olive oil consumption on primary end points for cardiovascular disease.

#### 7.2.3. Red Wine

Wine has been part of the human culture for more than 3000 years, serving dietary and socioreligious functions [305]. Several prospective studies have consistently demonstrated that weak/moderate red wine consumption (one to two drinks per day) is strongly associated with a lower incidence of cardiovascular diseases compared with teetotal or occasional alcohol consumption. The cardiovascular benefits of wine are likely due to combined, additive, or perhaps synergistic effects of alcohol and other wine components (mainly resveratrol and other polyphenolic compounds) on atherogenesis, coagulation, and fibrinolysis [305, 306]. Improved vascular function was shown in several experimental models of human diseases after administration of either red wine or its constituents and it was observed that the mechanisms involved in beneficial effects of natural polyphenols include also a reduction of platelet aggregation [307], increased high density lipoprotein (HDL) concentration [308], reduced LDL oxidation and concentration [307, 309, 310], and improvement of antioxidant defence system [311, 312]. Besides antioxidant properties, which play a significant role in cardioprotection, red wine polyphenols affect vascular function via modulation of NO bioavailability [313, 314]. Red wine compounds prevented metabolic and cardiovascular alterations in obese rats [310], improved endothelial-mediated dilatation and plasma NO level in hypercholesterolemic rabbits [307, 315], and restored endothelial function in deoxycorticosterone acetate-salt hypertensive rats [316]. In rats with fully developed NO-deficient hypertension, administration of red wine compounds produced a greater blood pressure decrease and improved endothelial functions [307].

Resveratrol, natural polyphenol found in grapes and grape products, including wine, as well as other sources [317], also decreased the gene expression of the potent vasoconstrictor ET-1 [305, 318]. Corder et al. [319] showed that red wine is capable of reducing ET-1 synthesis leading again to enhanced vasodilation. Our research group observed that treatment with resveratrol of type I diabetic rats may induce increase in adiponectin, the main adipokine secreted by fat deposits, that is strictly associated with a significant decrease in circulating ECs and EC fragmentations and a significant increase in PECAM-1 positive cells; moreover, we observed that resveratrol treatment may decrease ICAM-1 and VCAM-1 and caspase-3 activity in ECs, while increasing eNOS activity [320].

In humans, red wine and red wine compounds reduced blood pressure and improved forearm endothelial functions and NO production in young healthy individuals [321]. Similarly, a decrease of blood pressure in association with elevation of plasma NO was observed after both red wine and red wine compounds consumption in men at high cardiovascular risk [307, 322]. Additionally, treatment with red wine was found to reduce the susceptibility of LDLs to oxidation and to improve endothelium-dependent vasodilatation in patients with coronary heart disease [307, 323]. On the other hand, Andrade et al. [324] observed increased endothelial-mediated dilatation after short-term red wine consumption only in hypercholesterolemic, but not in hypertensive or healthy subjects [307]. However, in a study comparing the effect of water, red wine, beer, and vodka in healthy young subjects Huang et al. [325] found that only red wine affected endothelial function and significantly increased plasma levels of NO. Moreover, it is important to remember that, regarding the biological mechanisms linking endothelial function to moderate wine intake, it has been reported that alcohol *per se* can induce a marked increase in NO synthesis in primary cultures of bovine aortic ECs and human umbilical ECs, sustained by a rapid increase of eNOS protein and mRNA expression levels. In particular, both eNOS protein and mRNA increase by nearly twofold within 3 hours and gradually decline after 0.1% ethanol ingestion, but the increased levels of mRNA persist up to 24 hours [305, 326]. A 20-hour treatment of human umbilical vein ECs with an alcohol-free red wine polyphenol extract also led to a concentration-dependent increase in NO release, associated with an up to twofold increase of human eNOS promoter activity. Remarkably, although polyphenol extracts from wines of specific origin and grape cultivars vary strongly in their individual activity, when averaged, the activity cannot be attributed to a specific grape cultivar or growing area [305, 327]. Cuevas and colleagues [328] observed that moderate red wine consumption counteracts endothelial dysfunction induced by a high fat western diet administered to healthy men. The effects of ethanol and polyphenols on NO and consequently on endothelial function are very important, since endothelial function is an early indicator of atherosclerosis and vessel damage [316, 329, 330]. Moderate red wine consumption improved neovascularization and blood flow recovery after ischemia in hypercholesterolemic mice and had a positive effect on EPCs number and functional activity [331, 332]. Huang et al. [325] reported that red wine consumption by healthy subjects enhanced circulating EPC levels and improved EPC functions by modifying NO bioavailability. These studies support a modulatory effect of ethanol and/or polyphenols on EPC that may be antiatherogenic. Moreover, in a single blind crossover study, Whelan et al. [333] reported that the consumption of 4 mL/kg of either red or white wine with a light meal acutely improved brachial endothelial-mediated dilatation after 6 hours, whereas blood-alcohol levels had returned to baseline values by this time; the magnitude of this effect on endothelial function was substantially similar after both red and white wines. In healthy subjects, ingestion of 250 to 500 mL of red wine or dealcoholized red wine increased, in some, but not all studies, flow-mediated vasodilatation as assessed in the brachial artery by plethysmography [334–336].

It is still unclear whether the beneficial effects of red wine intake can be attributed to any specific type of grape and therefore any single wine source cannot be considered better than any other and it remains to be determined whether the reported benefits of red wine are based on one or more socioeconomic confounders. Accordingly, the beneficial effects of red wine intake in human health should be better defined and additional research works are required before any firm recommendation can be made to abstainers to initiate a light to moderate consumption of red wine [305]. Therefore, until more studies are conducted in order to elucidate this matter, it is not safe to advise consumption of a glass of red wine, as it is not clear if it would benefit or harm endothelial function in the immediate postprandial state [314].

#### 7.2.4. Antioxidants Supplementation

*Tempol*. Due to the central role of oxidative stress in development of cardiovascular diseases, many studies have been conducted over the past 20 years to investigate the potential use of antioxidants or mimetic of endogenous antioxidants, such as vitamins C and E, to reduce cardiovascular alterations [272].

Administration of tempol, a cell permeable SOD mimetic, has shown improvements in diabetes associated microvascular complications, such as nephropathy and retinopathy [59, 337]. In addition, tempol restores endothelial vasodilatation in large conduit vessels of alloxan-induced diabetic rabbits [58, 336]. Tempol increases endothelium-dependent vasodilatation in arteries from hypertensive animals, most likely through a lowering of ROS, but other mechanisms also appear to contribute to the effect [295].

MN40403, another highly specific nonpeptide SOD mimetic, was able to reverse endothelial dysfunction *ex vivo* by targeting NADPH oxidase-mediated superoxide production at aorta level of apolipoprotein E-deficient mice [58, 338].

*Vitamins C and E*. It has been detected that vitamins C and vitamin E, water, and lipid soluble antioxidants, respectively, produce beneficial effects on endothelial functions by modulating the downregulation of eNOS expression [339]. Moreover, the deleterious effects of postprandial hypertriglyceridemia on endothelial-dependent vasodilation can be counteracted by the simultaneous administration of vitamins C and E [243, 340, 341]. Maio et al. [342] investigated the acute effects of arterial vitamin C infusion on forearm blood flow in response to acetylcholine (a NO-dependent vasodilator) in 190 dipper and nondipper hypertensive patients (in dipper patients, but not in nondipper patients, blood pressure drops during night period). The authors found that vitamin C treatment improved blood flow following acetylcholine administration in both nondipper and dipper patients, but particularly in dipper ones. This observation supports and confirms the hypothesis that vitamin C improves endothelial function in hypertensive patients with impaired NO physiology. Vitamin C intake has also been shown to increase muscular blood flow during exercise in older adults and this effect is associated with improved endothelium-dependent vasodilatation mainly due to increased NO availability via eNOS [272, 343, 344].

The antioxidant effects of vitamins C and E are well established. However, studies both *in vitro* [345, 346] and *in vivo* [347, 348] have shown that antioxidant vitamins, particularly vitamin E, can have a paradoxical prooxidant effect when administrated under conditions of normal or low basal oxidative stress [349]. Indeed, the general trend towards increased cardiovascular mortality has opened a serious debate about the safety of vitamin E supplementation in high risk individuals. Further evidence about the efficacy of antioxidant vitamins is awaited from other ongoing trials of vitamin E alone or in combination with other antioxidants. Trying to explain the failure of vitamin E, it has been suggested that antioxidant therapy may require a longer treatment period because its primary mechanism of action is the prevention of new lesion formation [350].

*Melatonin*. Melatonin, an endogenously produced indoleamine, is a remarkably functionally pleiotropic molecule [351] which functions as a highly effective antioxidant and free radical scavenger [352, 353]. Endogenously produced and exogenously administered melatonin has known beneficial actions on the cardiovascular system [354, 355]. In particular, the antioxidant action of melatonin and its possible interaction with EDHF may contribute to blood pressure lowering effect, which was observed even when the NO pathway was inhibited [356]. However, melatonin seems to increase NO levels either through the stimulation of NO production and/or the prevention of coupling to the superoxide anion radical [357]. In addition melatonin was able to modulate *in vitro* acetylcholine-induced relaxation or phenylephrine-induced vasoconstriction of aortic rings of aging rats [358, 359] and New Zealand rabbits, respectively, in an endothelium-dependent manner [360]. Nevertheless, the mechanisms of these antihypertensive effects of melatonin are actually not completely understood.

Melatonin also may inhibit endothelium-derived adhesion molecules formation, reduce fatty acids infiltration in the intimal layer [361], and neutralize free radicals [362, 363]. Moreover, melatonin was demonstrated to prevent tissue injury and structural and functional alterations in the vasculature induced by cigarette smoking. Our research group observed and confirmed that melatonin minimized the damage induced by nicotine, reestablished the physiological balance between vasodilatation (increasing eNOS) and vasoconstriction (decreasing ET-1), induced antioxidant enzymes, and downregulated adhesive molecules on ECs [46, 220, 221].

## 8. Conclusions

Actually, compelling evidences indicate that an increased consumption of correct diet containing nutritive and nonnutritive compounds may contribute to the improvement of the quality of life by delaying onset and reducing the risk of cardiovascular diseases and, in particular, the development of endothelial dysfunction. In this context, wine, tea, fruits, vegetables, and olive oil received much attention, because they are particularly rich in natural antioxidants.

However, a better understanding of the mechanism(s) underlying EC dysfunction during cardiovascular physiopathology is a prerequisite for effective pharmacological and nonpharmacological interventions and treatments.

In conclusion, the proposal that antioxidants may ameliorate endothelial dysfunction is very interesting and promising, but further studies are needed to better understand the mechanisms that underline the biological effect of healthy life style.

## Figures and Tables

**Table 1:** High endothelial venules features with respect to normal venules, modified from Miyasaka and Tanaka (2004) [12].

	Normal venules	High endothelial venules
Endothelium	Flat	Tall and plump
Basal lamina	Thin	Thick
Perivascular sheath	Scant	Prominent
PECAM-1	Moderate	Moderate
ICAM-1	Very weak/absent	High
VE-cadherin	Moderate	Moderate

PECAM-1: platelet/endothelial cell adhesion molecule-1; ICAM-1: intercellular adhesion molecule-1; VE-cadherin: vascular endothelial-cadherin.
